# Whole Blood Mycobacterial Growth Assays for Assessing Human Tuberculosis Susceptibility: A Systematic Review and Meta-Analysis

**DOI:** 10.3389/fimmu.2021.641082

**Published:** 2021-05-11

**Authors:** Jeroen Bok, Regina W. Hofland, Carlton A. Evans

**Affiliations:** ^1^ Department of Infectious Disease, Imperial College London, London, United Kingdom; ^2^ Innovation for Health and Development (IFHAD), Laboratory of Research and Development, Universidad Peruana Cayetano Heredia, Lima, Peru; ^3^ Innovacion Por la Salud Y el Desarrollo (IPSYD), Asociación Benéfica PRISMA, Lima, Peru; ^4^ Department of Pulmonology and Tuberculosis, University Medical Center Utrecht, Utrecht, Netherlands

**Keywords:** tuberculosis, mycobacterial growth assay, mycobacterial growth inhibition assay, MGIA, susceptibility, risk

## Abstract

**Background:**

Whole blood mycobacterial growth assays (WBMGA) quantify mycobacterial growth in fresh blood samples and may have potential for assessing tuberculosis vaccines and identifying individuals at risk of tuberculosis. We evaluated the evidence for the underlying assumption that *in vitro* WBMGA results can predict *in vivo* tuberculosis susceptibility.

**Methods:**

A systematic search was done for studies assessing associations between WBMGA results and tuberculosis susceptibility. Meta-analyses were performed for eligible studies by calculating population-weighted averages.

**Results:**

No studies directly assessed whether WBMGA results predicted tuberculosis susceptibility. 15 studies assessed associations between WBMGA results and proven correlates of tuberculosis susceptibility, which we divided in two categories. Firstly, WBMGA associations with factors believed to reduce tuberculosis susceptibility were statistically significant in all eight studies of: BCG vaccination; vitamin D supplementation; altitude; and HIV-negativity/therapy. Secondly, WBMGA associations with probable correlates of tuberculosis susceptibility were statistically significant in three studies of tuberculosis disease, in a parasitism study and in two of the five studies of latent tuberculosis infection. Meta-analyses for associations between WBMGA results and BCG vaccination, tuberculosis infection, tuberculosis disease and HIV infection revealed consistent effects. There was considerable methodological heterogeneity.

**Conclusions:**

The study results generally showed significant associations between WBMGA results and correlates of tuberculosis susceptibility. However, no study directly assessed whether WBMGA results predicted actual susceptibility to tuberculosis infection or disease. We recommend optimization and standardization of WBMGA methodology and prospective studies to determine whether WBMGA predict susceptibility to tuberculosis disease.

## Introduction

Tuberculosis (TB) is estimated to make more than ten million people ill and to kill 1.4 million people each year globally (1). A quarter of the world population are believed to have latent TB infection (LTBI), in >90% of whom antimycobacterial immunity is expected to indefinitely prevent progression to TB disease. Several risk factors for progression from exposure to LTBI to active TB disease have been identified (2), but reliable predictors are lacking (3). Risk stratification, assessment of vaccines and other interventions aiming to reduce TB susceptibility are all complicated by the variable and often long delay from infection to disease and by difficulty determining TB exposure, infection and disease (4, 5). Consequently, there is an urgent need for *in vitro* assays to predict *in vivo* TB susceptibility.

Whole blood mycobacterial growth assays (WBMGA) aim to measure *in vitro* growth of mycobacteria in fresh blood samples. They are functional assays that, instead of focusing on a single immune marker, assess the combined effects of a range of factors such as immune mechanisms that influence mycobacterial growth *in vitro*. WBMGA have gained interest for TB vaccine testing, where pre- and post-vaccination assays may provide information about the efficacy of vaccine candidates, predicting individuals at risk of TB disease (6, 7). The underlying assumption is that if *in vitro* an individual’s blood allows greater mycobacterial growth then this finding predicts that individual to be at greater risk of developing TB infection or disease i.e., *in vivo* TB susceptibility.

In addition to WBMGA, mycobacterial growth assays have been developed and assessed using purified peripheral blood mononuclear cells (PBMC), purified macrophages, and bronchoalveolar lavage cells (6). In the current systematic review, we focused on WBMGA because of several advantages it offers compared to the PBMC-based mycobacterial growth assay: 1. the simplicity of WBMGA increases feasibility in the resource-constrained settings where most TB occurs (8); 2. whole-blood assays reduce the artefactual effects of cell-isolation procedures; and 3. the WBMGA is the *in vitro* approach that appears to best represent the complexities of *in vivo* responses, including the role of hemoglobin, neutrophilic granulocytes, antibodies and complement, which may explain the disagreement in results between WBMGA and equivalent assays using purified PBMC (9, 10).

Two main types of WBMGA have been used. Firstly, in the BCG lux assay, recombinant luminescent mycobacteria (BCG lux) are inoculated in diluted whole blood and a mycobacterial growth rate is calculated by measuring emitted light at the time of inoculation versus after incubation (11). Secondly, in the mycobacterial growth inhibition tube (MGIT) assay (12), mycobacteria are cultured in diluted whole blood, after which the mycobacteria are isolated and inoculated into BACTEC (Becton and Dickinson, Sparks, USA) MGIT culture tubes to assess time to mycobacterial detection, indicative of mycobacterial growth. WBMGA have used different blood supplements; infection with various *M. tuberculosis* strains and both wild-type or genetically modified BCG; incubation for 72-96 hours; and diverse outcome measures (e.g., mycobacterial time to culture positivity and mycobacterial bioluminescence indicating metabolism).

The central premise of a useful WBMGA is that mycobacterial growth measured *in vitro* predicts the *in vivo* risk of developing TB infection or active TB disease. Recently, the technical details of diverse WBMGA (and mycobacterial growth assays based on peripheral blood mononuclear cells) were reviewed (6). Our current review aims to extend these findings to determine what, if any evidence exists that human WBMGA results *in vitro* predict risk of TB *in vivo*. We aimed to include all types of human participants, interventions, comparisons, outcomes, and study designs (PICOS) with relevance to our objective (13).

## Methods

### Search Strategy and Selection Criteria

**Figure 1:**
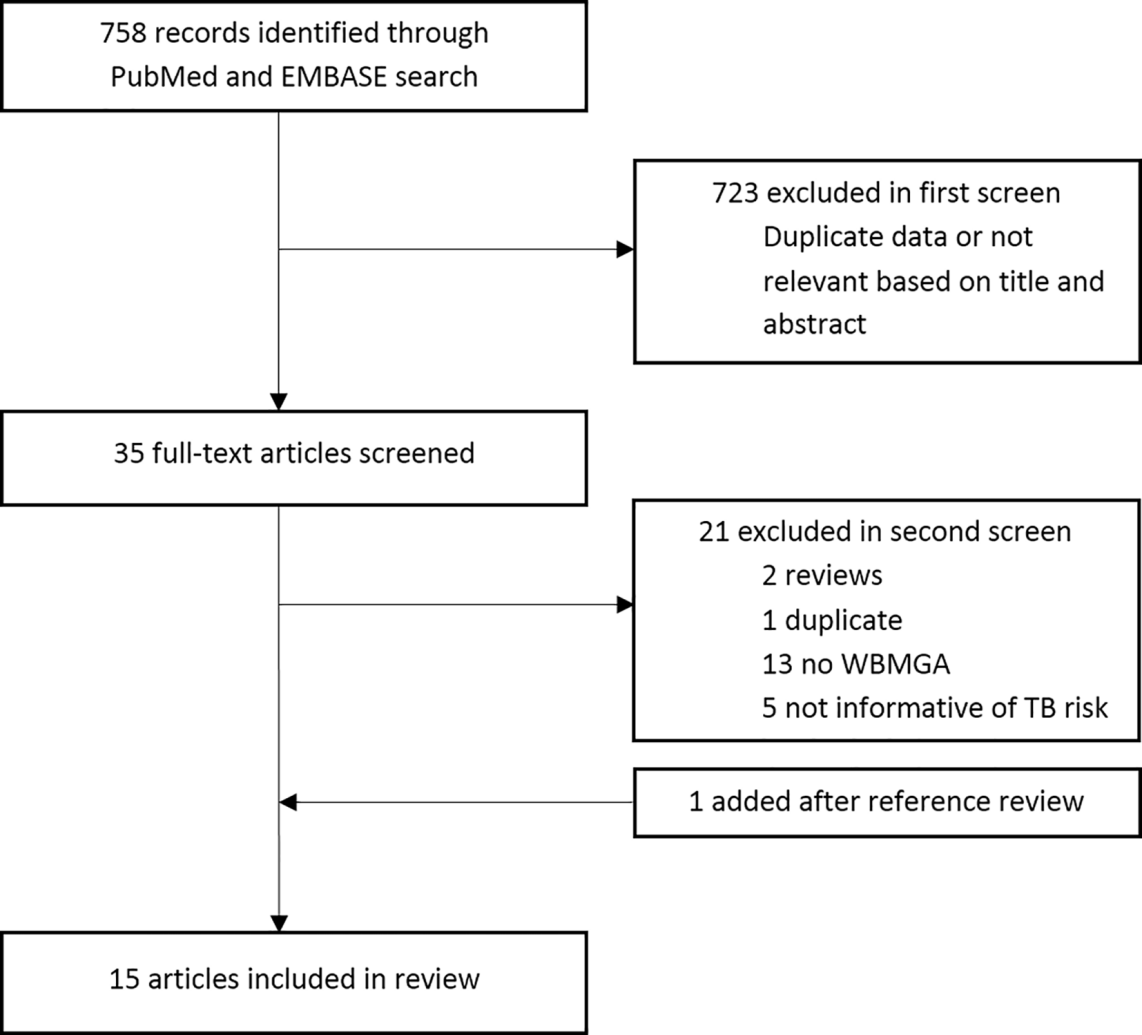
Flow chart of paper selection.

The search strategy is available at this link: http://www.ifhad.org/wp-content/uploads/2019/03/WBMGA_review_search_strategy.pdf. The systematic review protocol is available at this link: http://www.ifhad.org/wp-content/uploads/2019/03/WBMGA_review_protocol.pdf. The systematic review and meta-analysis registration is available at this link: http://www.ifhad.org/wp-content/uploads/2019/03/Systematic_review__meta-analysis_registration_submitted_to_PROSPERO.pdf. This review followed the PRISMA statement for reporting systematic reviews and meta-analyses (13). PubMed and EMBASE were searched until 25^th^ June 2020. References cited by these publications and reviews were searched. Inclusion criteria were: peer-reviewed, English-language publications that described cross-sectional, case-control, or cohort studies using WBMGA to study mycobacterial growth in human blood in relation to risk of TB infection; risk of TB disease; established or possible TB risk factors. JB and CAE reviewed potentially relevant publication titles, then abstracts and finally full-text publications for eligibility (**Figure 1**). Quality of the included studies was evaluated by JB and RH using a quality assessment tool from the National Heart, Lung, and Blood Institute (NHLBI), leading to an overall rating for the quality of each study of “good”, “fair”, or “poor” (14). Although derived for larger scale observational and cohort studies, this quality assessment tool seemed to be the best available option considering our inclusion criteria. Discrepancies were resolved through discussion.

### Data Analysis and Synthesis of Findings

WBMGA results, study characteristics and methodology were extracted from each publication and categorized by factors known to decrease or likely to affect TB susceptibility by JB and CE. WBMGA results were extracted as published, regardless of calculation or methodological differences.

**Figure 2:**
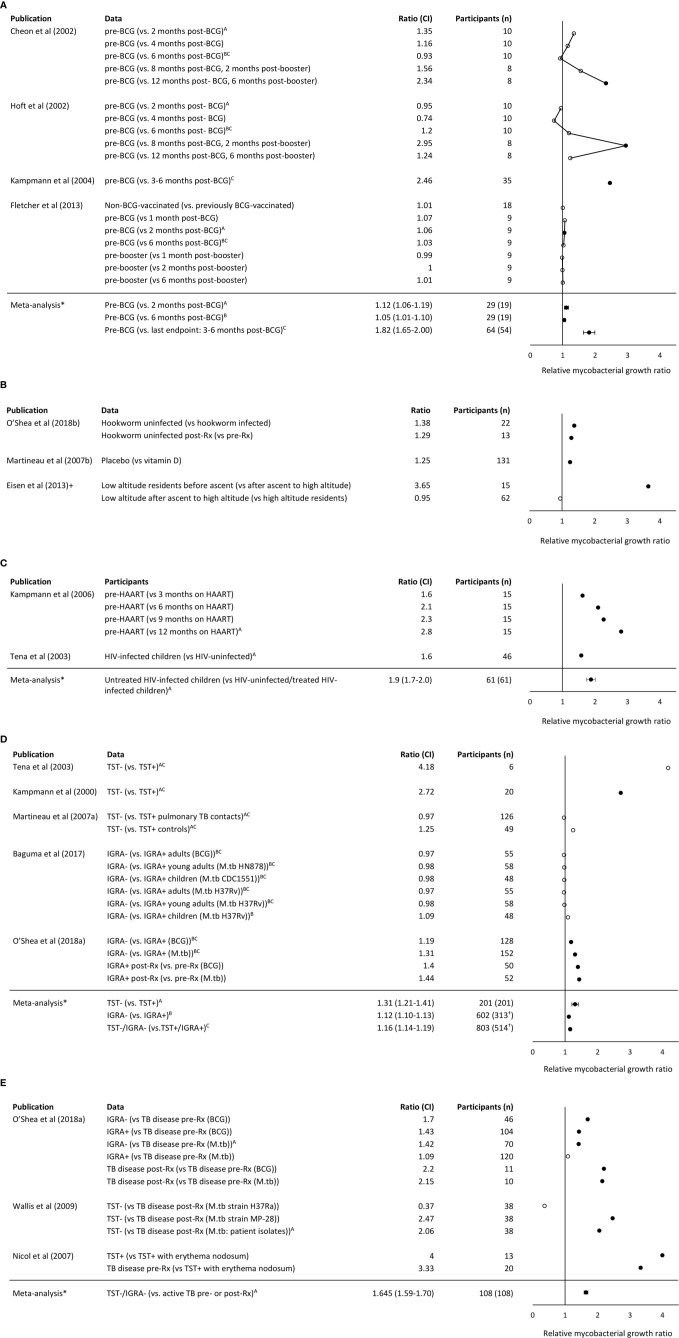
**(A)** Relative mycobacterial growth ratios of comparisons made in studies of BCG vaccination. **(B)** Relative mycobacterial growth ratios of comparisons made in studies of parasitism, vitamin D, and altitude, respectively. +Note the Eisen et al. (8) considered growth relative to control samples to adjust for altitude effects on mycobacterial growth. **(C)** Relative mycobacterial growth ratios of comparisons made in studies of HIV and its treatment. **(D)** Relative mycobacterial growth ratios of comparisons made in studies of TB infection. ^†^Approximation of population **(E)** Relative mycobacterial growth ratios of comparisons made in studies of TB disease. Note that higher relative mycobacterial growth ratio indicates greater mycobacterial growth so may be interpreted as implying relative susceptibility to mycobacterial infection in the participants listed without parentheses (compared with the participants listed in parentheses). Filled circles indicate P <0. 05. Meta-analysis mean and confidence interval methodology are explained in the Methods. BCG indicates Bacille Calmette Guerin. IGRA indicates the Interferon- γ release assay. *Comparisons included in the meta-analysis are marked with the corresponding letter **(A–C)**.

To allow comparison and synthesis of WBMGA results between different studies, ratios of one study group (e.g., pre-vaccination) versus the other (e.g., post-vaccination) were calculated for each of the main findings of the publications, generating relative mycobacterial growth ratios that are presented in **Figures 2A–E**.

When different WBMGA methodologies were used concurrently to assess a patient then the level of agreement between the methodologies was assessed with scatter plots and Pearson correlation coefficients.

### Meta-Analysis

Because of heterogeneity in statistical methods and lack of availability of participant-level data, standard deviations/errors could not be reliably estimated for each of the relative mycobacterial growth ratios that we calculated. Consequently, frequently used meta-analysis techniques incorporating study variances were impossible. Instead, for comparable studies we report averages of the relative mycobacterial growth ratios that we calculated weighted according to the number of participants in each study.

The equation used to calculate the weighted means was:

$$\bar{x}=\frac{\sum_{i=1}^{n}\left(x_{i}\cdot w_{i}\right)}{\sum_{i=1}^{n}w_{i}}$$

The equation used to calculate the standard error of weighted means was: 

$$\sigma_{x}={\left(\sqrt{\sum_{i=1}^{n}w_{i}}\right)}^{-1}$$

The standard errors of these weighted averages indicate the variation between individual studies and could not assess the variation within each study. These calculations used the R package “Hmisc” (15).

Heterogeneity was assessed visually with a histogram showing the log_10_ relative mycobacterial growth ratios in individual studies, indicating potential publication bias. Because the variance of each relative mycobacterial growth ratio was unknown, a conventional funnel plot could not be made. We therefore generated what we termed a pseudo-funnel plot of the log_10_ of the weighted means of relative mycobacterial growth ratios graphed against their standard errors, indicating potential publication bias in the weighted averages that we calculated.

## Results

### Results of Search

No prospective studies were found directly comparing WBMGA results with risk of TB infection or TB disease. Therefore, this review is limited to indirect evidence of studies testing associations between WBMGA results and factors believed to affect TB susceptibility. Fifteen articles meeting these criteria were included (**Figure 1**). A distinction was made between: (A) factors with consensus that they decrease TB susceptibility; and (B) factors likely affecting TB susceptibility but that lack consensus on whether they would increase or decrease susceptibility.

#### A. Factors Decreasing TB Susceptibility

**Table 1A** shows study results grouped according to the following factors believed to decrease TB susceptibility: BCG vaccination; vitamin D; altitude; and HIV negativity/therapy, all of which are summarized immediately below.

**Table 1A:** Overview of factors believed to decrease TB susceptibility and their association with less mycobacterial growth in WBMGA.

Category	Publication	Study group vs comparator	Bacteria^†^	P-value
TB risk	–	No studies predicting risk of infection or disease	NA	NA
BCG vaccination	Cheon et al. (16)	After primary vaccination (vs pre-vaccination)	BCG-lux^	NS
		After booster (vs pre-vaccination)	BCG-lux^	*
	Hoft et al. (17)	After primary vaccination (vs pre-vaccination)	BCG-lux	NS
		After booster (vs pre-vaccination)	BCG-lux	*
	Kampmann et al. (18)	After primary vaccination (vs pre-vaccination)	BCG-lux	*
	Fletcher et al. (19)	Previously vaccinated (vs unvaccinated)	BCG	NS
		After primary vaccination (vs pre-vaccination)	BCG	*
		After booster (vs pre-booster)	BCG	NS
Vitamin D supplementation	Martineau et al. (20)	Vitamin D supplemented (vs placebo)	BCG-lux	*
Altitude	Eisen et al. (8)	High- (vs low-) altitude residents at high altitude	BCG-lux	NS
		Before (vs after) ascent for low altitude residents	BCG-lux	*
HIV sero-negativity /therapy	Kampmann et al. (21)	After starting HAART treatment (vs pre-HAART)	BCG-lux	*
	Tena et al. (22)	HIV-uninfected (vs HIV-infected children (without HAART))	BCG-lux	*

^†^Growth of BCG-lux mycobacteria is measured using a BCG-lux assay, expect in the study by Cheon, where an MGIT assay was used.

*Any comparison was statistically significant.

NS, Not statistically significant comparison; NA, Statistical testing not available.

##### BCG Vaccination

**Figure 3:**
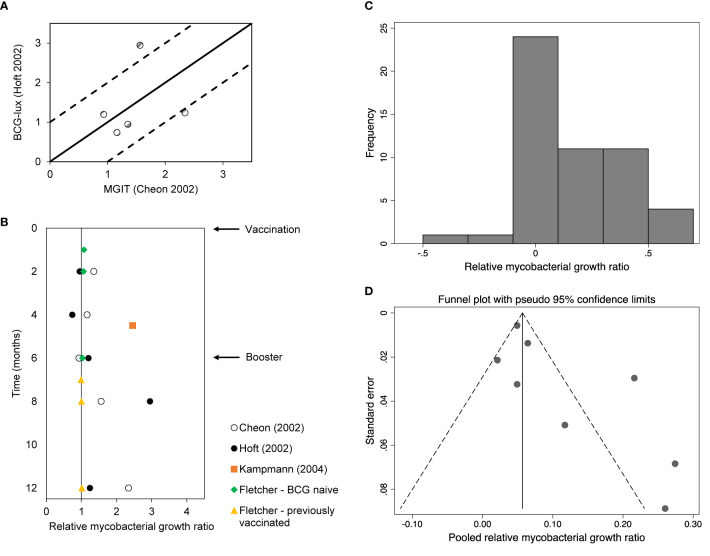
**(A)** Relative mycobacterial growth (ratios) of BCG vaccination studies using the same population but different assays. The solid line represents no difference between assay results. The dotted lines represent a 2-fold difference between assay results. **(B)** Relative mycobacterial growth (ratios) of BCG vaccination studies per month post-vaccination. **(C)** Histogram of log_10_ of relative mycobacterial growth ratios. Note this refers to the ratios as presented in **Figures 2A–E**. **(D)** Pseudo-funnel plot (see *Methods*).

BCG vaccination can offer protection of 60-80% against severe disseminated childhood TB, whereas protection against pulmonary TB varied considerably between studies (23). In the present review, three studies were identified that compared WBMGA pre- versus post-BCG-vaccination (**Figure 2A**). The BCG-lux technique demonstrated significantly decreased mycobacterial growth two months after secondary (8 months after primary) BCG vaccination in adults, but no significant effects persisted later (17). Concurrently the same blood samples (personal communication with Dr. Daniel Hoft) were tested with the MGIT technique, showing significantly decreased mycobacterial growth only six months after secondary (12 months after primary) BCG vaccination (16). The differences in relative mycobacterial growth at different time points between these studies are illustrated in **Figure 3A**, with a more than twofold difference at two time points. Significantly reduced mycobacterial growth in adults was reported only after primary vaccination of a cohort of BCG-naïve adults (although this depended on the statistical method) but no difference after secondary vaccination of a cohort of adults who had been vaccinated more than six months before enrolment (19). In the same study no difference in mycobacterial growth was found between the previously BCG-vaccinated versus the non-BCG-vaccinated groups at baseline. Reduced mycobacterial growth was also reported after neonatal BCG-vaccination (18). In **Figure 3B**, relative mycobacterial growth at different time points post-BCG vaccination are compared across all included studies.

##### Vitamin D

Low serum levels of vitamin D have been associated with an increased risk of TB disease (24). In the only study identified that analyzed vitamin D and WBMGA, in a randomized controlled trial a single dose of a vitamin D significantly reduced mycobacterial growth compared to placebo (**Figure 2B**) (20).

##### Altitude

High altitude is associated with lower risk of TB infection and disease (25, 26) and decreased mycobacterial growth was reported in low-altitude residents after ascent to high altitude, sufficient for there to be no difference between recently ascended individuals and permanent high-altitude residents (**Figure 2B**) (8).

##### HIV Negativity/Therapy

HIV infection is one of the strongest risk factors for progression to active TB disease (27). Two studies were identified that investigated WBMGA in relation to HIV infection (**Figure 2C**). Higher mycobacterial growth in HIV-infected children (without highly active antiretroviral therapy, HAART) was reported compared to HIV-uninfected children (22). Similarly, a significant decline in mycobacterial growth was reported after starting HAART in HIV-infected children (21).

#### B. Factors Likely Affecting TB Susceptibility

**Table 1B** shows study results grouped according to the following factors likely to affect TB susceptibility: TB infection, TB disease, and parasitism.

**Table 1B:** Overview of results of factors that may affect TB susceptibility and their association with less mycobacterial growth in WBMGA.

Category	Publication	Study group vs comparator	Bacteria	P-value
TB infection	Tena et al. (22)	TST+ (vs TST-)	BCG-lux	NA
	Kampmann et al. (11)	TST+ (vs TST-)	BCG-lux	*
	Martineau et al. (9)	TST+ (vs TST-)	BCG-lux	NS
	Baguma et al. (28)	IGRA+ (vs IGRA-)	BCG H37Rv HN878 CDC1551	NS
	O’Shea et al. (7)	IGRA+ (vs IGRA-)	BCG M.tb	**
		IGRA+ pre-Rx (vs IGRA+ post-Rx)	BCG M.tb	**
TB disease		TB disease (vs IGRA-)	BCG M.tb	**
		TB disease (vs IGRA+)	BCG M.tb	*
		TB disease pre-Rx (vs cured TB disease)	BCG M.tb	**
	Wallis et al. (29)	Cured TB disease (vs TST-)	Own$ MP28 H37RA	*
	Nicol et al. (30)	Erythema nodosum/TST+ (vs TB disease)	BCG-lux	*
Parasitism	O’Shea et al. (31)	Hookworm infected (vs uninfected)	H37Rv	*
		Hookworm infected pre- (vs post-) Rx	H37Rv	*

Own$ indicates the M. tuberculosis strain that caused the participant’s disease.

*Any comparison was statistically significant.

**All of multiple comparisons were statistically significant.

NS, Not statistically significant comparison; NA, Statistical testing not available; IGRA, indicates the Interferon- γ release assay.

##### TB Infection

Five studies were identified that analyzed the association between WBMGA and TB infection status, i.e., absence of infection indicated by negative tuberculin skin test (TST) and/or Interferon- γ release assay (IGRA) results versus TB infection (positive TST and/or IGRA) (**Figure 2D**). Three of these studies compared TST-positive versus TST-negative populations. Lower mycobacterial growth was reported in TST-positive versus TST-negative individuals, although statistical significance was not reported (22). Decreased mycobacterial growth was reported in TST-positive adults versus TST-negative adults (11). No significant difference in mycobacterial growth was found comparing TST-positive versus TST-negative adult contacts of patients diagnosed with pulmonary TB in a study designed to assess the role of neutrophils in host resistance to mycobacterial infection (9). Two other studies compared IGRA-positive and IGRA-negative populations. One found no significant difference in mycobacterial growth between IGRA-positive versus IGRA-negative children and adults in a high TB burden setting (28); the other reported significantly lower mycobacterial growth in IGRA-positive compared to IGRA-negative individuals and an increase in mycobacterial growth after treatment of IGRA-positive individuals (7).

##### TB Disease

Three studies reported the association between WBMGA and TB disease (**Figure 2E**). Patients with TB disease showed lowest mycobacterial growth, followed by IGRA-positive individuals, with highest mycobacterial growth in IGRA-negative individuals, although these associations were only observed when the mycobacteria used in the assay were BCG, not *M. tuberculosis* (7). Mycobacterial growth in patients cured of TB was less than TB-naïve individuals for two tested *M. tuberculosis* strains, but no significant difference was observed for a third *M. tuberculosis* strain (29). TST-positive children with erythema nodosum, a condition that was usually attributed TB infection in the setting of this study, showed less mycobacterial growth in WBMGA than children with active TB (30).

##### Parasitism

The evidence concerning the direction of the association between parasitism and risk of TB infection and TB disease is conflicting i.e. parasitism may be associated with decreased (31–33) or potentially (directly or indirectly through associated malnutrition) increased TB susceptibility (34, 35). One study was identified that examined the relation between helminth infections and WBMGA, which showed decreased mycobacterial growth in individuals with hookworm infection compared to hookworm-uninfected controls, which resolved after treatment of hookworm infection (**Figure 2B**) (31).

### Relative Mycobacterial Growth Ratios and Meta-Analysis

Relative mycobacterial growth ratios from the studies related to BCG vaccination, TB infection, TB disease and HIV infection are shown in **Figures 2A–D**, respectively, with each of these figures including meta- analyses. **Figure 2E** shows relative mycobacterial growth ratios from the studies related to parasitism, vitamin D and altitude; none of which were amenable to meta-analysis. The meta-analyses showed the following:

- Mycobacterial growth in WBMGA was significantly reduced 2-6 months after primary BCG vaccination (**Figure 2A**). The available data concerning BCG booster vaccination were not amenable to meta-analysis (see legend to **Figure 2A**).
- Mycobacterial growth was significantly less for TB-infected than for TB-uninfected populations (whether infection was assessed by TST or IGRA, **Figure 2B**).
- Mycobacterial growth was significantly less for patients with TB disease (whether before or after treatment) than for TB-uninfected people (TST- or IGRA-negative, **Figure 2C**).
- Mycobacterial growth was significantly less in relatively immunocompetent people (whether HIV-uninfected people or HIV-infected people receiving HAART) than untreated people with HIV-infection (**Figure 2D**).

The histogram depicting the log_10_ of the relative mycobacterial growth ratios (**Figure 3C**) and the pseudo-funnel plot (**Figure 3D**) are both skewed right, which may indicate publication bias.

### Study Characteristics and Assay Methodology

Study characteristics of the included studies and the WBMGA methodology that were used are presented in **Table 2** and **Table 3**, respectively. Assay controls were used in 53% (eight of 15) of the included studies. Considerable heterogeneity in population, setting and reported statistics were found (**Table 2**). Methodological characteristics comparing studies, including concentrations of mycobacterial inoculate and the use of controls, were diverse (**Table 3**).

**Table 2:** Study characteristics.

Publication	N	Participants	Setting	Study design	Reported statistic
Cheon et al. (16)	10	Healthy adults	St. Louis, USA	Longitudinal	Mean (standard deviation)
Hoft et al. (17)	10	Healthy adults	St. Louis, USA	Longitudinal	Median (50% range, non-outlier range)
Kampmann et al. (18)	35	Healthy neonates	Cape Town, South Africa	Longitudinal	Median (range)
Fletcher et al. (19)	18	Healthy adults	United Kingdom	Cross-sectional/longitudinal	Median (lowest of 25^th^ quartile, highest of 75^th^ quartile)
Martineau et al. (20)	131	Adult TB contacts	United Kingdom	Randomized controlled trial	Mean (confidence interval of group difference)
Eisen et al. (8)	62	Healthy adults	Lima, Peru (low altitude) Cusco, Peru (high altitude)	Cross-sectional/longitudinal	Median (interquartile range)
Kampmann et al. (21)	15	HIV-infected, BCG-vaccinated children	Cape Town, South Africa	Longitudinal	Median (range)
Tena et al. (22)	22 24	HIV-infected children HIV-uninfected children	Cape Town, South Africa	Cross-sectional	Median (range)
Kampmann et al. (11)	20	Healthy adults	United Kingdom	Cross-sectional	Median (range)
Martineau et al. (9)	126 49	Adult TB contacts Healthy adults	London, United Kingdom	Cross-sectional	Mean (standard deviation)
Baguma et al. (28)	161	BCG-vaccinated children and adults	Western Cape Province, South Africa	Cross-sectional	Median (interquartile range, range)
O’Shea et al. (7)	19 101 51	Active TB patients LTBI patients healthy adults	United Kingdom, various locations	Cross-sectional/longitudinal	Mean (standard deviation)
Wallis et al. (29)	32 6	Cured TB patients Healthy adults	Vitória, Brazil (TB patients) Newark, USA (controls)	Cross-sectional	Mean
Nicol et al. (30)	5 15 8	Children with erythema nodosum Children with active TB Healthy TST-positive children	Cape Town, South Africa	Cross-sectional	Median
O’Shea et al. (31)	22	Healthy adult migrants from Nepal	United Kingdom	Cross-sectional/longitudinal	Mean (standard deviation)

Note that ‘N’ indicates the study population (including those that did not complete follow-up, in cases where this is applicable). Also note that the order of the publications in this table, and in **Tables 3** and **4**, is consistent with **Table 1A** and **1B**.

**Table 3:** Assay methodology.

Publication	Growth calculation	Assay type	MOI	Concentration	Volume per assay (ml)	Media added pervolume of blood	Incubation time (h)	Replicates	Assay controls
Cheon et al. (16)	Δlog_10_CFU = log_10_(final) – log_10_(initial)	MGIT	NR	10,000 CFU/ml (100,000 RLU/ml)	0.6	1:1 RPMI + glutamine + 25 mM HEPES	96	2	Simultaneous direct mycobacterial inoculation of MGIT tube
Hoft et al. (17)	Mycobacterial inhibition index = (RLU at pre-BCG day 3 or day 4/RLU at pre-BCG day 0)/(Post-BCG day 3 or day 4 RLU/post-BCG day 0 RLU)	BCG-lux	NR	10,000 CFU/ml (100,000 RLU/ml)	1	1:2 RPMI	96	3	None reported
Kampmann et al. (18)	Growth ratio = RLU at T_96_/RLU at T_0_	BCG-lux	NR	1,000,000 CFU/ml (10,000,000 RLU/ml)	1	1:1 RPMI	96	3	None reported
Fletcher et al. (19)	Δlog_10_ CFU per day = log((CFU of sample at T_96/_CFU of control at T_96_)/4)	MGIT	NR	150 CFU in 600 μl	0.6	1:1 RPMI	96	2	Simultaneous direct mycobacterial inoculation of MGIT tube (duplicate)
Martineau et al. (20)	Luminescence ratio = RLU at T_24_ or T_96_/RLU at T_0_	BCG-lux	1	300,000 CFU/ml	1	1:1 RPMI + 2 mM glutamine + 25 mM HEPES	96	3	None reported
Eisen et al. (8)	(RLU at T_96_ – RLU at T_0_)/RLU of culture broth	BCG-lux	30	10,000 CFU/ml (100,000 RLU/ml), 200 ul blood in each of quadruplet tests	1	1:1 RPMI + 1% HEPES	72	4	Supplemented 7H9 broth; plasma
Kampmann et al. (21)	Growth ratio = RLU at T_96_/RLU at T_0_	BCG-lux	NR	1,000,000 CFU/ml (10,000,000 RLU/ml)	1	1:1 RPMI	96	3	None reported
Tena et al. (22)	Growth ratio = RLU at T_96_/RLU at T_0_	BCG-lux	NR	1,000,000 CFU/ml (10,000,000 RLU/ml)	1	1:1 RPMI	96	3	None reported
Kampmann et al. (11)	Growth ratio = (RLU at T_96_ – RLU at T_0_)/(RLU at T_0_)	BCG-lux	NR	10,000 CFU/ml (100,000 RLU/ml)	1	1:1 RPMI + 1% L-glutamine and heparin	96	3	Plasma
Martineau et al. (9)	Luminescence ratio = RLU at T_96_/RLU at T_0_	BCG-lux	1	300,000 CFU/ml	1	1:1 RPMI + 2 mM glutamine + 25 mM HEPES	96	3	None reported
Baguma et al. (28)	Δlog_10_ CFU = log_10_(final) – log_10_(initial)	MGIT	NR	8,500 – 2,4000 CFU/ml	0.6	1:1 RPMI	96	2	Simultaneous direct mycobacterial inoculation of MGIT tube
O’Shea et al. (7)	Growth ratio = log_10_(CFU of sample/CFU of control)	MGIT	NR	150 CFU/600 μl	0.6	1:1 RPMI containing 10% pooled human serum + 2 mM L-glutamine and 25 mM HEPES	96	2	Simultaneous direct mycobacterial inoculation of MGIT tube (duplicate)
Wallis et al. (29)	Δlog_10_CFU = log_10_(final) – log_10_(initial)	MGIT	NR	10,000 CFU/ml (100,000 RLU/ml)	0.6	1:1 tissue culture medium	72	2/1*	Simultaneous direct mycobacterial inoculation of MGIT tube
Nicol et al. (30)	Growth ratio = RLU at T_96_/RLU at T_0_	BCG-lux	NR	1,000,000 CFU/ml (10,000,000 RLU/ml)	1	1:1 RPMI	96	3	None reported
O’Shea et al. (31)	Growth ratio = log_10_(CFU of sample/CFU of control)	MGIT	NR	150 CFU/600 μl	0.6	1:1 RPMI containing 10% pooled human serum + 2 mM L-glutamine and 25 mM HEPES	96	2	Simultaneous direct mycobacterial inoculation of MGIT tube (duplicate)

Note MOI indicates the multiplicity of infection stated as the number of monocytes estimated to be present in the assay per colony forming unit of mycobacteria. RLU, relative light units; GI, growth index; CFU, colony forming units; BCG, bacille Calmette-Guerrin; MOI, Multiplicity of Infection, mycobacteria per macrophage; *Duplicate in Brazil, single in USA.

### Study Quality

**Table 4** shows the result of a study quality evaluation using a standardized quality assessment tool developed by NHLBI. Two of the included studies received a good rating, ten received a fair rating, and three received a poor rating.

**Table 4:** Study quality.

Publication	Objective^1^	Population^2^	Participation^3^	Recruitment^4^	Sample size^5^	Exposure measurement^6^	Timeframe^7^	Exposure levels^8^	Exposure validity^9^	Exposure assessed^10^	Outcome validity^11^	Blinding^12^	Loss to follow-up^13^	Adjustment confounders^14^	Rating^b^
Cheon et al. (16)	Yes	No	NA	NR	No	Yes	Yes	Yes	Yes	NA	NA	NR	NA	No	Fair
Hoft et al. (17)	Yes	No	NA	NR	No	Yes	Yes	Yes	Yes	NA	NA	NR	NA	No	Fair
Kampmann et al. (18)	Yes	No	NR	NR	No	Yes	No	NA	Yes	NA	NA	NR	NA	No	Fair
Fletcher et al. (19)	Yes	No	NR	NR	No	Yes	Yes	Yes	No	NA	NA	NR	NA	No	Poor
Martineau et al. (20)	Yes	Yes	Yes	Yes	Yes	Yes	Yes	No	Yes	No	No	Yes	No	No	Good
Eisen et al. (8)	Yes	No	NR	NR	No	Yes	Yes	No	Yes	NA	NA	NR	NA	No	Fair
Kampmann et al. (21)	Yes	Yes	NR	Yes	No	Yes	Yes	NA	Yes	No	NA	NR	NA	No	Fair
Tena et al. (22)	Yes	No	NR	NR	No	Yes	Yes	NA	No	No	NA	NR	NA	No	Fair
Kampmann et al. (11)	Yes	No	NR	NR	No	Yes	Yes	NA	Yes	No	NA	NR	NA	No	Fair
Martineau et al. (9)^c^	NA	Yes	Yes	Yes	No	Yes	Yes	NA	Yes	No	NA	NR	NA	Yes	Fair^c^
Baguma et al. (28)	Yes	No	NR	NR	No	Yes	Yes	NA	Yes	No	NA	NR	NA	No	Fair
O’Shea et al. (7)	Yes	No	NR	NR	No	Yes	Yes	Yes	Yes	No	NA	NR	NA	No	Good
Wallis et al. (29)	Yes	No	NR	No	No	Yes	Yes	NA	Yes	No	NA	NR	NA	No	Poor
Nicol et al. (30)	Yes	No	NR	NR	No	Yes	Yes	NA	No	No	NA	NR	NA	No	Poor
O’Shea et al. (31)	Yes	Yes	NR	Yes	No	Yes	Yes	NA	Yes	No	NA	NR	NR	No	Fair

a Numbers refer to the following questions that are part of the National Heart, Lung, and Blood Institute’s (NHLBI) Quality Assessment Tool for Observational Cohort and Cross-Sectional Studies:

1. Was the research question or objective in this paper clearly stated?

2. Was the study population clearly specified and defined?

3. Was the participation rate of eligible persons at least 50%?

4. Were all the subjects selected or recruited from the same or similar populations (including the same time period)? Were inclusion and exclusion criteria for being in the study prespecified and applied uniformly to all participants?

5. Was a sample size justification, power description, or variance and effect estimates provided?

6. For the analyses in this paper, were the exposure(s) of interest measured prior to the outcome(s) being measured?

7. Was the timeframe sufficient so that one could reasonably expect to see an association between exposure and outcome if it existed?

8. For exposures that can vary in amount or level, did the study examine different levels of the exposure as related to the outcome (e.g., categories of exposure, or exposure measured as continuous variable)?

9. Were the exposure measures (independent variables) clearly defined, valid, reliable, and implemented consistently across all study participants?

10. Was the exposure(s) assessed more than once over time?

11. Were the outcome measures (dependent variables) clearly defined, valid, reliable, and implemented consistently across all study participants?

12. Were the outcome assessors blinded to the exposure status of participants?

13. Was loss to follow-up after baseline 20% or less?

14. Were key potential confounding variables measured and adjusted statistically for their impact on the relationship between exposure(s) and outcome(s)?

Possible answers: Yes; No; CD, cannot determine; NA, not applicable; NR, not reported.

b Possible ratings: good, fair, poor.

c Rating of this applies to quality of data extracted for this systematic review, not to quality of main study.

### Comparison of BCG-lux and MGIT Assay Results

**Figure 3A** shows differences between the results of BCG-lux and MGIT assays performed concurrently on the same whole blood samples. The Pearson correlation coefficient of the BCG-lux and MGIT assay results, presented as mycobacterial growth ratios, was 0.19 (R^2^ = 0.037). Two of five data points showed a more than two-fold difference in growth ratio.

### Heterogeneity of BCG Vaccination Study Results

**Figure 3B** illustrates the heterogeneity of WBMGA results of BCG vaccination studies at different time points post-vaccination.

## Discussion

This systematic review and meta-analysis assessed evidence that low mycobacterial growth in WBMGA predicted lower TB susceptibility. This demonstrated that less mycobacterial growth *in vitro* in WBMGA was indeed usually significantly associated with factors believed to reduce peoples’ TB susceptibility *in vivo*. Factors that are likely to affect TB susceptibility, but that lack consensus on whether they would increase or decrease susceptibility also generally showed significant and consistent associations with WBMGA results. This implies potential WBMGA value for clinical risk stratification and evaluation of TB vaccines, despite considerable clinical, laboratory and statistical heterogeneity across the included studies.

Developing biomarkers to predict TB risk is a priority for global TB elimination (1). Promising progress has been made recently, including identification of RNA and metabolic signatures (36, 37) and clinical risk scores (4, 5, 38). Growth assays aim to functionally assess host capacity to control infections, such as for example, malaria growth assays that predicted disease risk by a specific strain of *Plasmodium falciparum* (39). The emphasis of mycobacterial growth assay research has been on vaccine efficacy and immune mediator studies, with limited information on prospective risk of TB disease (6). Data on the relation between WBMGA and TB risk is thus limited to indirect evidence, which was assessed in this review.

By quantifying mycobacterial growth *in vitro*, WBMGA may be representative of the balance between factors influencing progression of mycobacterial infection versus containment of the infection through host antimycobacterial immunity. It is generally hypothesized that less mycobacterial growth in WBMGA *in vitro* implies immune restriction of mycobacteria and hence less TB susceptibility, i.e. a lower risk of TB infection or TB disease *in vivo* (6). In the current review, we found that WBMGA studies of factors believed to reduce TB susceptibility i.e., BCG vaccination, HIV negativity/therapy, vitamin D supplementation, and ascent to altitude largely supported this hypothesis. Although each of the included studies on BCG vaccination showed a significant association with WBMGA results, the time from vaccination until a significant inhibition of mycobacterial growth varied considerably, potentially because of methodological and population heterogeneity. Furthermore, although the protective efficacy of BCG vaccination against severe childhood TB is considerable, the protection it offers against pulmonary TB is variable and likely dependent on various host-dependent and environmental factors, including variations in exposure to environmental mycobacteria and BCG strains, confounding comparability and interpretation of these studies (23, 40). It is noteworthy that all WBMGA studies of BCG vaccination used BCG *in vitro*; thus assessment of the potential effect of BCG vaccination on *M. tuberculosis* growth in whole blood *in vitro* is awaited. It is unknown whether lower mycobacterial growth *in vitro* post-BCG vaccination implies long-term protection against TB disease rather than a temporary strengthening of adaptive antimycobacterial immunity or trained innate immunity (41).

The extent to which TB exposure and latent TB infection (LTBI) may affect susceptibility to TB disease caused by TB reactivation versus reinfection is debated (42). Currently the main tests to diagnose LTBI are TST and IGRA, which have limitations including indirectly assessing immunological memory rather than directly assessing actual infection (43). These tests only weakly predict the risk of subsequent TB disease and their results are influenced by factors including nutritional status and other causes of immunodeficiency (44, 45). An association might be expected between more mycobacterial growth in WBMGA (potentially implying greater TB susceptibility), leading to higher likelihood of LTBI, consistent with the proven association between LTBI and increased future TB risk. However, this hypothesis was not supported by any of the included studies. Rather, two of the five included studies reported significant associations and both indicated the opposite association. Specifically, less mycobacterial growth in WBMGA (potentially implying less TB susceptibility) was found in people with LTBI, despite their proven increased future risk of TB disease, possibly because mycobacterial replication in the host may provoke an immune response inhibiting mycobacterial growth in WBMGA (7). It has been suggested that this provides information about an individual’s position on the spectrum of LTBI, following the increasing recognition that LTBI represents a diverse group ranging from those who may have completely cleared the infection to those with actively replicating *M. tuberculosis* without clinical symptoms (46). If WBMGA results coincide with this spectrum, they may help to inform risk stratification of progression to active TB (7). The results of the included study by O’Shea do appear to imply this, but it is not specified whether patients with active TB were already receiving treatment, which may influence *in vitro* mycobacterial growth (7). These findings may all be explained by the hypothesis that latent TB infection or TB disease both cause immune activation that reduces TB susceptibility (as indicated by reduced mycobacterial growth in WBMGA), reducing the risk that a new exposure to TB will cause super-infection, re-infection or subsequent TB disease. This integrating hypothesis is supported by some epidemiological data and animal experimentation and should be the focus of future research (45, 47).

Helminth infections have geographical overlap with LTBI and TB disease. Some helminths including hookworm infection suppress the antimycobacterial immune responses measured by TST and IGRA, and this suppression is reversible with anthelminthic treatment (48, 49). This could be a direct effect of helminths that are known to cause some forms of immunosuppression and anergy (50), or might be caused indirectly by helminth infections causing malnutrition, which also suppresses some measures of antimycobacterial immunity (34). Thus, helminth infections may suppress antimycobacterial immunity sufficiently to increase TB susceptibility (50), causing helminth infections to be associated with more mycobacterial growth in WBMGA. However, there is contrary evidence that helminth infections may instead stimulate antimycobacterial immunity (33) and the one study on helminths and WBMGA demonstrated that hookworm infection (but not other helminth species) was associated with less mycobacterial growth in WBMGA, which was reversible with hookworm treatment. There was some evidence for mediation by hookworm-induced eosinophilia (31). These seemingly contradictory findings may be explained by the complexity of antimycobacterial immunity: the antimycobacterial immunity measured by TST and IGRA may be distinct from the mediators assessed in the WBMGA.

A strength of this study that it is the first assessment of whether diverse studies suggest that WBMGA results predict TB risk. Limitations included the absence of direct evidence, so the included studies could not provide a direct answer to our research question. Another limitation was diversity: the profound variations in study design, methodology, statistical analysis, population and sample size in the studies that our systematic review identified confounded their comparison and synthesis by meta-analysis, and also complicated the assessment of study quality. Particularly concerning was the lack of controls in approximately half of the included studies. Variation in reported statistical methodology and failure of most of the included studies to publish their source data prevented us from calculating confidence intervals in our assessments of WBMGA results and forced us to calculate weighted average effect rather than using optimal meta-analysis techniques, limiting the precision of our meta-analyses.

After the literature search of this systematic review was finished, a study from The Gambia was published that would have met our inclusion criteria if it been published earlier and is noteworthy for two main methodological reasons (51). Firstly, this study used a novel auto-luminescent WBMGA, which allows for collecting smaller volume blood samples and serial measurement of luminescence without sample destruction. Secondly, WBMGA were used to assess pairs of highly TB-exposed children with discordant TST status, a novel study design that allows for comparison of individuals with a presumably similar level of TB exposure (51). This contrasts with the studies included in our review in which TB exposure could be a potential confounding factor. However, apart from these two novel methodological advances, the findings of this study were similar to the studies included in our review, demonstrating greater mycobacterial growth in uninfected children than in infected children. Thus, this recently published study does not alter the conclusions of our systematic review.

In conclusion, WBMGA results usually showed statistically significant associations with factors known or likely to affect TB susceptibility. However, these studies were diverse and there is a need for methodological standardization as well as a systematic assessment of reproducibility of WBMGA results, as has been done for PBMC-based assays (52). Importantly, prospective evaluations of whether WBMGA predict peoples’ risk of TB infection or disease are urgently needed, although these studies are likely to be slow and expensive because of the relatively low incidence of either outcome, the long interval over which these outcomes develop, and diagnostic difficulties that make the absence of TB infection or disease difficult to prove. Prospective studies should assess whether an optimized and standardized WBMGA may be useful for TB risk stratification or evaluation of new TB vaccine candidates.

## Data Availability Statement

All data presented in the study are included in the article, the links in the Methods section of the article, or the publications cited in the article. Further inquiries can be directed to the corresponding author.

## Author Contributions

JB, RH, and CE contributed to the conception of the study. JB and CE searched the data. JB and CE extracted the data. JB analyzed the data. JB, RH, and CE interpreted the data. JB, RH and CE prepared the manuscript. All authors contributed to the article and approved the submitted version.

## Funding

Funding is gratefully acknowledged from: the Wellcome Trust (awards 057434/Z/99/Z, 070005/Z/02/Z, 078340/Z/05/Z, 105788/Z/14/Z, and 201251/Z/16/Z); UK-AID DFID-CSCF; the Joint Global Health Trials Scheme (award MR/K007467/1) with funding from the UK Foreign, Commonwealth and Development Office, the UK Medical Research Council, the UK Department of Health and Social Care through the National Institute of Health Research (NIHR) and Wellcome; the STOP TB partnership’s TB REACH initiative funded by the Government of Canada and the Bill & Melinda Gates Foundation (awards W5_PER_CDT1_PRISMA and OPP1118545); CONCYTEC/ FONDECYT award code E067-2020-02-01 number 083-2020; and the charity IFHAD: Innovation For Health And Development.

## Conflict of Interest 

The authors declare that the research was conducted in the absence of any commercial or financial relationships that could be construed as a potential conflict of interest.

## References

[1] Global Tuberculosis Report 2020. World Health Organization. Available at: https://www.who.int/teams/global-tuberculosis-programme/tb-reports/global-tuberculosis-report-2020 (Accessed Feb. 24, 2021).

[2] Narasimhan P Wood J Macintyre CR Mathai D. Risk factors for tuberculosis. Pulm Med (2013) 2013:828939. 10.1155/2013/828939 23476764 PMC3583136

[3] Walzl G Ronacher K Hanekom W Scriba TJ Zumla A. Immunological biomarkers of tuberculosis. Nat Rev Immunol (2011) 11:343–54. 10.1038/nri2960 21475309

[4] Saunders MJ Wingfield T Tovar MA Baldwin MR Datta S Zevallos K. A score to predict and stratify risk of tuberculosis in adult contacts of tuberculosis index cases: a prospective derivation and external validation cohort study. Lancet Infect Dis (2017) 17:1190–9. 10.1016/S1473-3099(17)30447-4 PMC7611139 28827142

[5] Saunders MJ Tovar MA Collier D Baldwin MR Montoya R Valencia TR. Active and passive case-finding in tuberculosis-affected households in Peru: a 10-year prospective cohort study. Lancet Infect Dis (2019) 19:519–28. 10.1016/S1473-3099(18)30753-9 PMC6483977 30910427

[6] Tanner R O’Shea MK Fletcher HA McShane H. In vitro mycobacterial growth inhibition assays: A tool for the assessment of protective immunity and evaluation of tuberculosis vaccine efficacy. Vaccine (2016) 34:4656–65. 10.1016/j.vaccine.2016.07.058 27527814

[7] O’Shea MK Tanner R Müller J Harris SA Wright D Stockdale L. Immunological correlates of mycobacterial growth inhibition describe a spectrum of tuberculosis infection. Sci Rep (2018a) 8:14480. 10.1038/s41598-018-32755-x 30262883 PMC6160428

[8] Eisen S Pealing L Aldridge RW Siedner MJ Necochea A Leybell I. Effects of Ascent to High Altitude on Human Antimycobacterial Immunity. PLoS One (2013) 8:e74220. 10.1371/journal.pone.0074220 24058530 PMC3772817

[9] Martineau AR Newton SM Wilkinson KA Kampmann B Hall BM Nawroly N. Neutrophil-mediated innate immune resistance to mycobacteria. J Clin Invest (2007a) 117:1988–94. 10.1172/JCI31097 PMC1904316 17607367

[10] Tanner R O'Shea MK White AD Müller J Harrington-Kandt R Matsumiya M. The influence of haemoglobin and iron on *in vitro* mycobacterial growth inhibition assays. Sci Rep (2017) 7. 10.1038/srep43478 PMC5335253 28256545

[11] Kampmann B Gaora PÓ Snewin VA Gares M Young DB Levin M. Evaluation of Human Antimycobacterial Immunity Using Recombinant Reporter Mycobacteria. J Infect Dis (2000) 182:895–901. 10.1086/315766 10950786

[12] Wallis RS Palaci M Vinhas S Hise AG Ribeiro FC Landen K. A Whole Blood Bactericidal Assay for Tuberculosis. J Infect Dis (2001) 183:1300–3. 10.1086/319679 11262217

[13] Moher D Liberati A Tetzlaff J Altman DG Group TP. Preferred Reporting Items for Systematic Reviews and Meta-Analyses: The PRISMA Statement. PLoS Med (2009) 6:e1000097. 10.1371/journal.pmed.1000097 19621072 PMC2707599

[14] National Heart Lung and Blood Institute. Quality assessment tool for observational cohort and cross-sectional studies. (2014). Available at: https://www.nhlbi.nih.gov/health-topics/study-quality-assessment-tools (Accessed Feb. 24, 2021)

[15] Harrell FE. Hmisc: A package of miscellaneous R functions (2014). Available at: https://hbiostat.org/R/Hmisc/ (Accessed Jul. 1, 2020).

[16] Cheon S-H Kampmann B Hise AG Phillips M Song H-Y Landen K. Surrogate Marker of Immunity after Vaccination against Tuberculosis. Clin Vaccine Immunol (2002) 9:901–7. 10.1128/CDLI.9.4.901-907.2002 PMC120034 12093693

[17] Hoft DF Worku S Kampmann B Whalen CC Ellner JJ Hirsch CS. Investigation of the Relationships between Immune-Mediated Inhibition of Mycobacterial Growth and Other Potential Surrogate Markers of Protective Mycobacterium tuberculosis Immunity. J Infect Dis (2002) 186:1448–57. 10.1086/344359 12404160

[18] Kampmann B Tena GN Mzazi S Eley B Young DB Levin M. Novel Human In Vitro System for Evaluating Antimycobacterial Vaccines. Infect Immun (2004) 72:6401–7. 10.1128/IAI.72.11.6401-6407.2004 PMC522995 15501770

[19] Fletcher HA Worku S Kampmann B Whalen CC Ellner JJ Hirsch CS. Inhibition of Mycobacterial Growth In Vitro following Primary but Not Secondary Vaccination with Mycobacterium bovis BCG. Clin Vaccine Immunol (2013) 20:1683–9. 10.1128/CVI.00427-13 PMC3837779 23986316

[20] Martineau AR Wilkinson RJ Wilkinson KA Newton SM Kampmann B Hall BM. A single dose of vitamin D enhances immunity to mycobacteria. Am J Respir Crit Care Med (2007b) 176:208–13. 10.1164/rccm.200701-007OC 17463418

[21] Kampmann B Tena-Coki GN Nicol MP Levin M Eley B. Reconstitution of antimycobacterial immune responses in HIV-infected children receiving HAART. AIDS (2006) 20:1011–8. 10.1097/01.aids.0000222073.45372.ce 16603853

[22] Tena GN Young DB Eley B Henderson H Nicol MP Levin M. Failure to control growth of mycobacteria in blood from children infected with human immunodeficiency virus and its relationship to T cell function. J Infect Dis (2003) 187:1544–51. 10.1086/374799 12721934

[23] Roy A Eisenhut M Harris RJ Rodrigues LC Sridhar S Habermann S. Effect of BCG vaccination against Mycobacterium tuberculosis infection in children: systematic review and meta-analysis. BMJ (2014) 349. 10.1136/bmj.g4643 PMC4122754 25097193

[24] Nnoaham KE Clarke A. Low serum vitamin D levels and tuberculosis: a systematic review and meta-analysis. Int J Epidemiol (2008) 37:113–9. 10.1093/ije/dym247 18245055

[25] Vargas MH Furuya MEY Pérez-Guzmán C. Effect of altitude on the frequency of pulmonary tuberculosis. Int J Tuberc Lung Dis (2004) 4:1321–4. 15581199

[26] Saito M Wilkinson RJ Wilkinson KA Newton SM Kampmann B Hall BM. Comparison of altitude effect on Mycobacterium tuberculosis infection between rural and urban communities in Peru. Am J Trop Med Hygiene (2006) 75:49–54. 10.4269/ajtmh.2006.75.49 16837708

[27] Corbett EL Watt CJ Walker N Maher D Williams BG Raviglione MC. The growing burden of tuberculosis: global trends and interactions with the HIV epidemic. Arch Intern Med (2003) 163:1009–21. 10.1001/archinte.163.9.1009 12742798

[28] Baguma R Wilkinson RJ Wilkinson KA Newton SM Kampmann B Hall BM. Application of a whole blood mycobacterial growth inhibition assay to study immunity against Mycobacterium tuberculosis in a high tuberculosis burden population. PLoS One (2017) 12:e0184563. 10.1371/journal.pone.0184563 28886145 PMC5590973

[29] Wallis RS Vinhas S Janulionis E. Strain specificity of antimycobacterial immunity in whole blood culture after cure of tuberculosis. Tuberculosis (2009) 89:221–4. 10.1016/j.tube.2009.02.001 PMC2752959 19321387

[30] Nicol MP Kampmann B Lawrence P Wood K Pienaar S Pienaar D. Enhanced Anti-Mycobacterial Immunity in Children with Erythema Nodosum and a Positive Tuberculin Skin Test. J Invest Dermatol (2007) 127:2152–7. 10.1038/sj.jid.5700845 17460727

[31] O’Shea MK Fletcher TE Muller J Tanner R Matsumiya M Bailey JW. Human Hookworm Infection Enhances Mycobacterial Growth Inhibition and Associates With Reduced Risk of Tuberculosis Infection. Front Immunol (2018b) 9:2893. 10.3389/fimmu.2018.02893 30619265 PMC6302045

[32] du Plessis N Walzl G. “Helminth-M. Tb Co-Infection”. In: Horsnell W, editor. How Helminths Alter Immunity to Infection. New York, NY: Springer New York (2014). p. 49–74.

[33] Borelli V Vita F Shankar S Soranzo MR Banfi E Scialino G. Human Eosinophil Peroxidase Induces Surface Alteration, Killing, and Lysis of Mycobacterium tuberculosis. Infect Immun (2003) 71:605–13. 10.1128/IAI.71.2.605-613.2003 PMC145361 12540536

[34] Cegielski JP McMurray DN. The relationship between malnutrition and tuberculosis: evidence from studies in humans and experimental animals. Int J Tuberc Lung Dis (2004) 14:286–98. 15139466

[35] Elias D Mengistu G Akuffo H Britton S. Are intestinal helminths risk factors for developing active tuberculosis? Trop Med Int Health (2006) 11:551–8. 10.1111/j.1365-3156.2006.01578.x 16553939

[36] Zak DE Penn-Nicholson A Scriba TJ Thompson E Suliman S Amon LM. A blood RNA signature for tuberculosis disease risk: a prospective cohort study. Lancet (2016) 387:2312–22. 10.1016/S0140-6736(15)01316-1 PMC5392204 27017310

[37] Weiner J Maertzdorf J Sutherland JS Duffy FJ Thompson E Suliman S. Metabolite changes in blood predict the onset of tuberculosis. Nat Commun (2018) 9:5208. 10.1038/s41467-018-07635-7 30523338 PMC6283869

[38] Mandalakas AM Kirchner HL Lombard C Walzl G Grewal HMS Gie RP. Well-quantified tuberculosis exposure is a reliable surrogate measure of tuberculosis infection. The International journal of tuberculosis and lung disease (2012) 16(8):1033–39. 10.5588/ijtld.12.0027 10.5588/ijtld.12.0027 PMC11967560 22692027

[39] Rono J Färnert A Olsson D Osier F Rooth I Persson KEM. Plasmodium falciparum line-dependent association of *in vitro* growth-inhibitory activity and risk of malaria. Infect Immun (2012) 80:1900–8. 10.1128/IAI.06190-11 PMC3347460 22392930

[40] Fine PEM. Variation in protection by BCG: implications of and for heterologous immunity. Lancet (1995) 346:1339–45. 10.1016/S0140-6736(95)92348-9 7475776

[41] Joosten SA van Meijgaarden KE Arend SM Prins C Oftung F Korsvold GE. Mycobacterial growth inhibition is associated with trained innate immunity. J Clin Invest (2018) 128:1837–51. 10.1172/JCI97508 PMC5919803 29461976

[42] Esmail H Barry CE Young DB Wilkinson RJ. The ongoing challenge of latent tuberculosis. Philos Trans R Soc B: Biol Sci (2014) 369:20130437. 10.1098/rstb.2013.0437 PMC4024230 24821923

[43] Diel R Loddenkemper R Nienhaus A. Predictive value of interferon-γ release assays and tuberculin skin testing for progression from latent TB infection to disease state: a meta-analysis. Chest (2012) 142:63–75. 10.1378/chest.11-3157 22490872

[44] Rangaka MX Wilkinson KA Glynn JR Ling D Menzies D Mwansa-Kambafwile J. Predictive value of interferon-γ release assays for incident active tuberculosis: a systematic review and meta-analysis. Lancet Infect Dis (2012) 12:45–55. 10.1016/S1473-3099(11)70210-9 21846592 PMC3568693

[45] Andrews JR Noubary F Walensky RP Cerda R Losina E Horsburgh CR. Risk of Progression to Active Tuberculosis Following Reinfection With Mycobacterium tuberculosis. Clin Infect Dis (2012) 54:784–91. 10.1093/cid/cir951 PMC3284215 22267721

[46] Barry CE 3rd Boshoff HI Dartois V Dick T Ehrt S Flynn J. The spectrum of latent tuberculosis: rethinking the biology and intervention strategies. Nat Rev Microbiol (2009) 7:845–55. 10.1038/nrmicro2236 PMC4144869 19855401

[47] Cadena AM Hopkins FF Maiello P Carey AF Wong EA Martin CJ. Concurrent infection with Mycobacterium tuberculosis confers robust protection against secondary infection in macaques. PLoS Pathog (2018) 14:e1007305. 10.1371/journal.ppat.1007305 30312351 PMC6200282

[48] Elias D Wolday D Akuffo H Petros B Bronner U Britton S. Effect of deworming on human T cell responses to mycobacterial antigens in helminth-exposed individuals before and after bacille Calmette–Guérin (BCG) vaccination. Clin Exp Immunol (2001) 123:219–25. 10.1046/j.1365-2249.2001.01446.x PMC1905995 11207651

[49] Banfield S Pascoe E Thambiran A Siafarikas A Burgner D. Factors Associated with the Performance of a Blood-Based Interferon-γ Release Assay in Diagnosing Tuberculosis. PLoS One (2012) 7(6):e38556. 10.1371/journal.pone.0038556 22701664 PMC3373489

[50] Babu S Nutman TB. Helminth-Tuberculosis Co-Infection: an Immunologic Perspective. Trends Immunol (2016) 37:597–607. 10.1016/j.it.2016.07.005 27501916 PMC5003706

[51] Basu Roy R Sambou B Sissoko M Holder B Gomez MP Egere U. Protection against mycobacterial infection: A case-control study of mycobacterial immune responses in pairs of Gambian children with discordant infection status despite matched TB exposure. EBioMedicine (2020) 59:102891. 10.1016/j.ebiom.2020.102891 32675024 PMC7502674

[52] Tanner R Smith SG van Meijgaarden KE Giannoni F Wilkie M Gabriele L. Optimisation, harmonisation and standardisation of the direct mycobacterial growth inhibition assay using cryopreserved human peripheral blood mononuclear cells. J Immunol Methods (2019) 469:1–10. 10.1016/j.jim.2019.01.006 30710562 PMC7926177

